# Implicit data crimes: Machine learning bias arising from misuse of public data

**DOI:** 10.1073/pnas.2117203119

**Published:** 2022-03-21

**Authors:** Efrat Shimron, Jonathan I. Tamir, Ke Wang, Michael Lustig

**Affiliations:** ^a^Department of Electrical Engineering and Computer Sciences, University of California, Berkeley, CA 94720;; ^b^Department of Electrical and Computer Engineering, The University of Texas at Austin, Austin, TX 78712;; ^c^Department of Diagnostic Medicine, Dell Medical School, The University of Texas at Austin, Austin, TX 78712;; ^d^Oden Institute for Computational Engineering and Sciences, The University of Texas at Austin, Austin, TX 78712

**Keywords:** data crimes, inverse problem, big data, MRI, bias

## Abstract

Although open databases are an important resource in the current deep learning (DL) era, they are sometimes used “off label”: Data published for one task are used to train algorithms for a different one. This work aims to highlight that this common practice may lead to biased, overly optimistic results. We demonstrate this phenomenon for inverse problem solvers and show how their biased performance stems from hidden data-processing pipelines. We describe two processing pipelines typical of open-access databases and study their effects on three well-established algorithms developed for MRI reconstruction: compressed sensing, dictionary learning, and DL. Our results demonstrate that all these algorithms yield systematically biased results when they are naively trained on seemingly appropriate data: The normalized rms error improves consistently with the extent of data processing, showing an artificial improvement of 25 to 48% in some cases. Because this phenomenon is not widely known, biased results sometimes are published as state of the art; we refer to that as implicit “data crimes.” This work hence aims to raise awareness regarding naive off-label usage of big data and reveal the vulnerability of modern inverse problem solvers to the resulting bias.

## Significance

Public databases are an important resource for machine learning research, but their growing availability sometimes leads to “off-label” usage, where data published for one task are used for another. This work reveals that such off-label usage could lead to biased, overly optimistic results of machine-learning algorithms. The underlying cause is that public data are processed with hidden processing pipelines that alter the data features. Here we study three well-known algorithms developed for image reconstruction from magnetic resonance imaging measurements and show they could produce biased results with up to 48% artificial improvement when applied to public databases. We relate to the publication of such results as implicit “data crimes” to raise community awareness of this growing big data problem.

Public databases are an important driving force in the current deep learning (DL) revolution; ImageNet (1) is a well-known example. However, due to the growing availability of open-access data and the general hype around artificial intelligence, databases are sometimes used in an “off-label” manner: Data published for one task are used for different ones. Here we aim to show that such naive and seemingly appropriate usage of open-access data could lead to biased, overly optimistic results.

Biased performance of machine-learning models due to faulty construction of data cohorts or research pipelines recently has been identified for various tasks, including gender classification (2), COVID-19 prediction (3), and natural language processing (4). However, to the best of our knowledge, it has not been studied for inverse problem solvers. We address this gap by highlighting scenarios that lead to biased performance of algorithms developed for image reconstruction from undersampled MRI measurements; the latter is a real-world example of an inverse problem and a current frontier of DL research (5–13).

The MRI measurements are fundamentally acquired in the Fourier domain, which is known as k-space. Sub-Nyquist sampling is commonly applied to shorten the traditionally lengthy MRI scan time, and image reconstruction algorithms are used to recover images from the undersampled data (14–17). Therefore, the development of such algorithms ideally should be done using raw k-space data. However, the development of DL methods requires thousands of examples, and databases containing raw k-space data are scarce. To date, only a few databases offer such data (for example, refs. 18–22), whereas many more offer reconstructed and processed magnetic resonance (MR) images (for example, refs. 23–30). The latter offer images for postreconstruction tasks, such as segmentation and biomarker discovery. Nevertheless, due to their availability, they often are downloaded and used to synthesize “raw” k-space data using the forward Fourier transform; the synthesized data are then used for the development of reconstruction algorithms. We identified that this common approach could lead to undesirable consequences; the underlying cause is that the nonraw MR images are commonly processed using hidden pipelines. These pipelines, which are implemented by commercial scanner software or during database storage, include a full set or a subset of the following steps: image reconstruction, filtering, storage of magnitude data only (i.e., loss of the MRI complex values), lossy compression, and conversion to Digital Imaging and Communications in Medicine (DICOM) or Neuroimaging Informatics Technology Initative (NIFTI) formats. These reduce the data entropy. We aim to highlight that when modern algorithms are trained and evaluated using such data, they benefit from the data processing and, hence, tend to exhibit overly optimistic results compared to performance on raw, unprocessed data. Because this phenomenon is largely unknown, such biased results are sometimes published as state of the art without reporting the processing pipelines or addressing their effects. To raise community awareness of this growing problem, we coin the term “data crimes” to describe such publications, in reference to the more obvious “inverse crime” scenario (31) described next.

Bias stemming from the underlying data has been recognized previously in a few scenarios related to inverse problems. The term inverse crime describes a scenario in which an algorithm is tested using simulated data, and the simulation resonates with the algorithm such that it leads to improved results (31–35). Specifically, the authors of ref. 34 described an inverse crime as a situation where the same discrete model is used for simulating k-space measurements and reconstructing an MR image from them. They showed that compared with reconstruction from raw or analytically computed measurements, this leads to reduced ringing artifacts. A second example is evaluation of MRI reconstruction algorithms on real-valued magnitude images. In this case, k-space exhibits conjugate symmetry; hence, it is sufficient to use only about half of it for full image recovery. This symmetry often is leveraged in partial Fourier methods such as Homodyne (15) and projection onto convex sets (36), where additional steps are applied for recovery of the full complex data. However, neglecting the fact that the data are complex valued results in better conditioning due to the lower dimensionality of the inverse problem. This may lead to an obvious advantage when evaluating algorithms on such data as opposed to raw k-space data. However, to the best of our knowledge, inverse crimes have not been studied yet in the context of machine learning or public data usage.

Here we report on two subtle forms of algorithmic bias that have not been described in the literature yet and that are relevant to the current DL era. We show how they arise from two hidden data-processing pipelines that affect many open-access MRI databases: a commercial scanner pipeline and a JPEG data storage pipeline. To demonstrate these scenarios, we took raw MRI data and “spoiled” them with carefully controlled processing steps. We then used the processed datasets for training and evaluation of algorithms from three well-established MRI reconstruction frameworks: compressed sensing (CS) with a wavelet transform (37), dictionary learning (DictL) (38), and DL (39). Our experiments demonstrate that these algorithms yield overly optimistic results when trained and evaluated on processed data.

The main contributions of this work are fivefold. First, we reveal scenarios in which algorithmic bias of inverse problem solvers may arise from off-label usage of open-access databases and analyze them through large-scale statistics. Second, we find that CS, DictL, and DL algorithms are all prone to this form of subtle bias. While recent studies identified stability issues of MRI reconstruction algorithms (5, 40), here we identify a common vulnerability of canonical algorithms to data-related bias. Third, we demonstrate the potentially harmful impact of data crimes by showing that methods trained on processed data but applied to unprocessed data yield lower-quality image reconstruction in real-world scenarios. Fourth, our experiments reveal limited generalization ability of the studied algorithms. Finally, by introducing the concept of data crimes, we hope to raise community awareness of the growing problem of bias stemming from off-label usage of open-access data.

## Data Crimes

In this section, we lay out the framework for our experiments.

### Data Crime I: Zero-Padded k-Space Data.

**Fig. 1.**
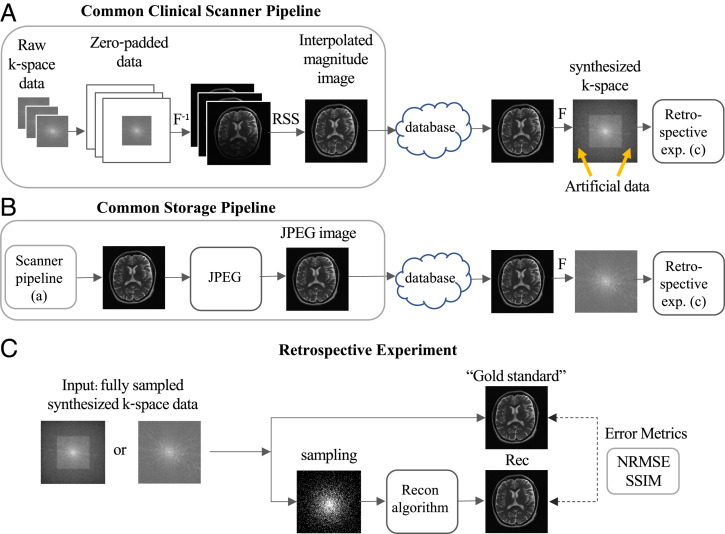
Data crimes: how retrospective subsampling of processed data leads to biased results. (*A*) A common data processing pipeline often implemented inside commercial MRI scanners includes k-space zero padding, application of the inverse Fourier transform, and coil combination via an RSS step. The output image, which is interpolated and nonnegative, is stored in a database. In data crime I, this image is later used for synthesizing new k-space data; this yields artificial data in previously zero-padded areas. (*B*) A common data storage pipeline includes JPEG compression. In data crime II, the compressed image is later used for retrospective experiments. (*C*) Standard research pipelines commonly involve retrospective subsampling of fully sampled k-space data. In the data crimes scenarios, the fully sampled data are based on processed data; hence, image reconstruction algorithms benefit from the early processing. Moreover, because the gold standard image is based on the same processed data as the reconstructed one, error metrics become blind to the processing and, therefore, also are prone to bias.

We first consider a data-processing pipeline that is implemented inside many commercial MRI scanners for reconstructing the scanner output (i.e., the MR image). The k-space data are typically acquired using a multicoil array, and the pipeline includes the following steps (Fig. 1*A*): 1) image interpolation, implemented by zero padding the raw multicoil k-space data; 2) application of the inverse discrete Fourier transform; and 3) multicoil image combination via a square root sum of squares (RSS) step. Notice that although the acquired data are complex valued, the RSS step produces a magnitude image. The scanner output, therefore, is an interpolated real-valued nonnegative image; this is the type of image most prevalent in online MRI databases.

Let us assume that the scanner image is later downloaded and used to synthesize new k-space data, with the aim of using those data to train a reconstruction algorithm. The synthesized k-space has two interesting features not originally present: It is larger than the original raw k-space (due to the zero padding), and it has nonzero values everywhere (due to the nonlinear RSS step). In other words, the true data now lie in the k-space center, whereas artificial data appear in its periphery (Fig. 1*A*, yellow arrows). However, because this k-space looks fully sampled, it is considered to be “ground truth” and used for algorithm development.

A research pipeline commonly used in the development of MRI reconstruction algorithms is based on retrospective subsampling, in which sub-Nyquist sampling is simulated using a binary sampling mask and applied to a fully sampled k-space (Fig. 1*C*). In the studied data crime scenario, such retrospective subsampling is applied to the synthesized k-space, which includes artificial data. Common subsampling masks typically are based on variable density (VD) sampling schemes, which sample the center of k-space more densely than its periphery. These VD schemes are used because they produce incoherent aliasing artifacts that can be removed by sparsity-promoting, optimization-based reconstruction algorithms (37). Importantly, because k-space was zero-padded earlier in the pipeline, application of a VD mask to the entire area of the synthesized k-space results in higher effective sampling density of the true k-space data.

**Fig. 2.**
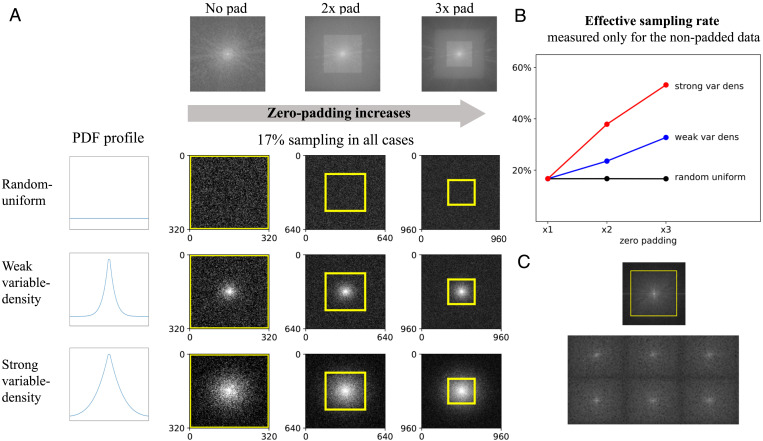
An experiment demonstrating how retrospective subsampling of k-space synthesized from processed data leads to increased effective sampling density of the true k-space data. (*A*) Subsampling masks were generated for different combinations of zero-padding factors (*Left* to *Right*) and subsampling schemes (*Top* to *Bottom*). The masks were generated from symmetric 2D PDFs (profiles displayed) with 17% sampling in all cases. The regions covering the original nonpadded k-space data are marked with yellow boxes. Notice that the zero padding squashes the original data to the center, so when a VD scheme is used, those data are sampled with an increased rate. (*B*) The effective sampling rate, which is the subsampling rate inside the original k-space area (i.e., inside the yellow boxes in *A*), versus the zero-padding rate. Notice that for the variable-density schemes, the effective rate is much higher than the global rate (17%) and may rise above 55%. (*C*) Real-world examples for k-space data generated from MR images found in public open-access databases (23, 24) show evidence of zero padding (the yellow box is our estimation). These experiments and examples indicate that training algorithms using data from public databases could lead to increased effective sampling.

To demonstrate this, we performed the following experiment: We generated subsampling masks for combinations of three zero-padding factors and three subsampling schemes (Fig. 2*A*). All the masks included a global sampling rate of 17%, which corresponds to an acceleration factor of *R* = 6; this rate is measured for the full k-space area. Then we measured the effective sampling rate, which we defined as the sampling rate in the nonpadded areas only (yellow boxes in Fig. 2*A*), and plotted it against the zero-padding rate (Fig. 2*B*). The results indicate that for VD subsampling (both weak VD and strong VD), the effective sampling rate is much higher than the global rate. In the case of 2× zero padding, which is often applied by default in commercial scanners, the measured effective rates are 24% (*R* = 4.1) and 38% (*R* = 2.6) for weak and strong VD sampling, respectively. These are much larger than the global rate of 17% (*R* = 6). Nevertheless, because researchers often miss this subtle effect, only the global rate is reported. It is then claimed that algorithms are suitable for reconstruction for a subsampling rate that is much larger than the one used in practice.

In summary, our experiment demonstrates that when processed data are retrospectively subsampled with a VD scheme, there is increased sampling density of “true” data. In the experiments described in *Results*, we demonstrate that this gives rise to overly optimistic algorithm performance.

### Data Crime II: JPEG-Compressed Data.

The second studied pipeline involves JPEG compression of the scanner image (Fig. 1*B*). Such compression is commonly used to reduce storage footprint, and it is sometimes applied as part of the DICOM data-saving pipeline, which is highly prevalent for storage of medical images. To demonstrate the JPEG effect, here we neglect the zero-padding scenario, although the two effects are sometimes combined. In the scenario of data crime II, the JPEG-compressed image is stored in an online database and later downloaded and used to synthesize a new k-space, which is used for algorithm development (Fig. 1*C*). However, because JPEG compression reduces the data entropy, using JPEG data in retrospective subsampling experiments leads to improved reconstruction fidelity. We aim to show that this leads to an artificial improvement of image reconstruction algorithms.

## Results

We studied the effects of the hidden data-processing pipelines by simulating those pipelines using carefully controlled processed data. Implementation details are provided in *Materials and Methods*. Preliminary results of this work were presented at the Annual Meeting of International Society of Magnetic Resonance in Medicine (ISMRM) (41).

### Data Crime I.

**Fig. 3.**
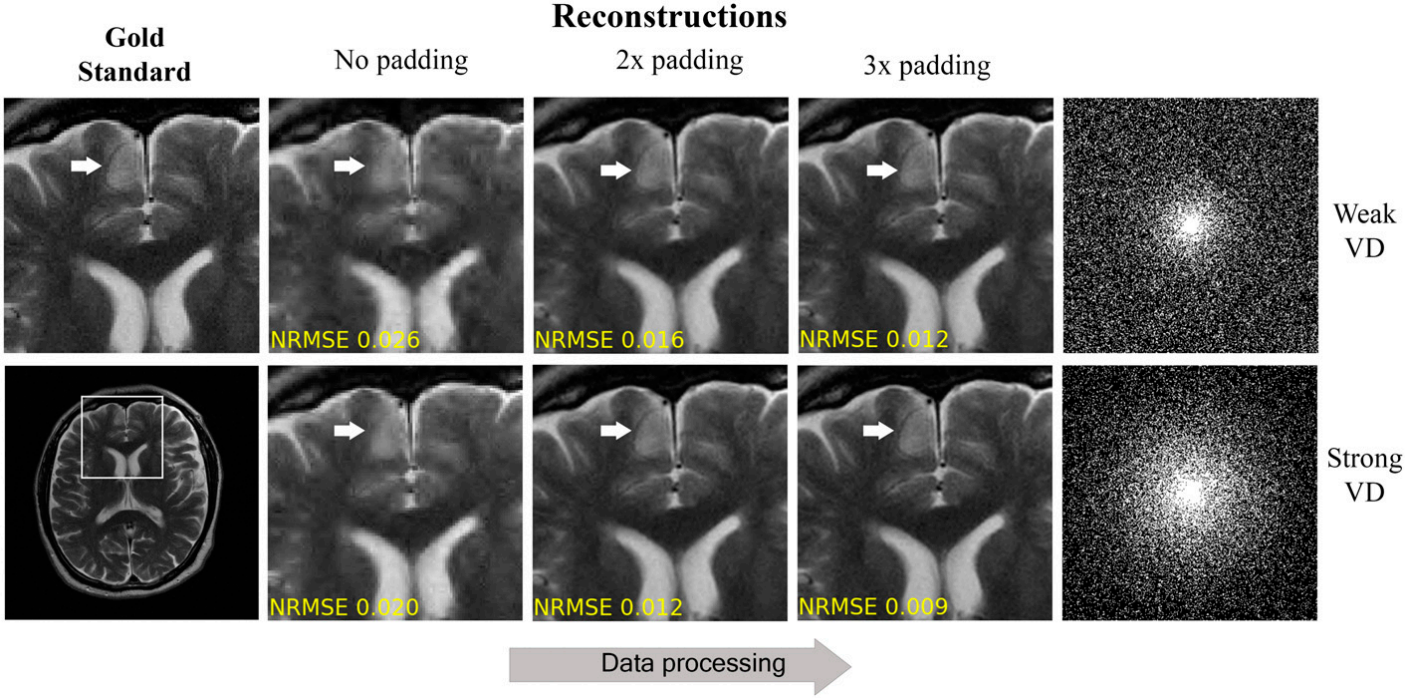
Example for data crime I: CS reconstructions from retrospectively subsampled k-space of processed images. Notice how the reconstruction quality improves, both visually and in terms of NRMSE, with the zero-padding (processing) extent. This improvement is completely artificial; it stems from the coupling of early data processing and retrospective subsampling, which leads to increased sampling of true nonpadded data (as illustrated in Fig. 2). The artificial improvement is more significant when the sampling is stronger around the k-space center (*Bottom*; strong VD).

The first experiment examined the effect of the commercial scanner processing pipeline (Fig. 1*A*) on the CS algorithm. The results show that this algorithm produces increasingly sharper reconstructions as the k-space zero-padding factor grows, for both weak and strong VD sampling schemes (Fig. 3). This effect was reflected by an artificial reduction of the normalized rms error (NRMSE) as a function of the zero padding.

**Fig. 4.**
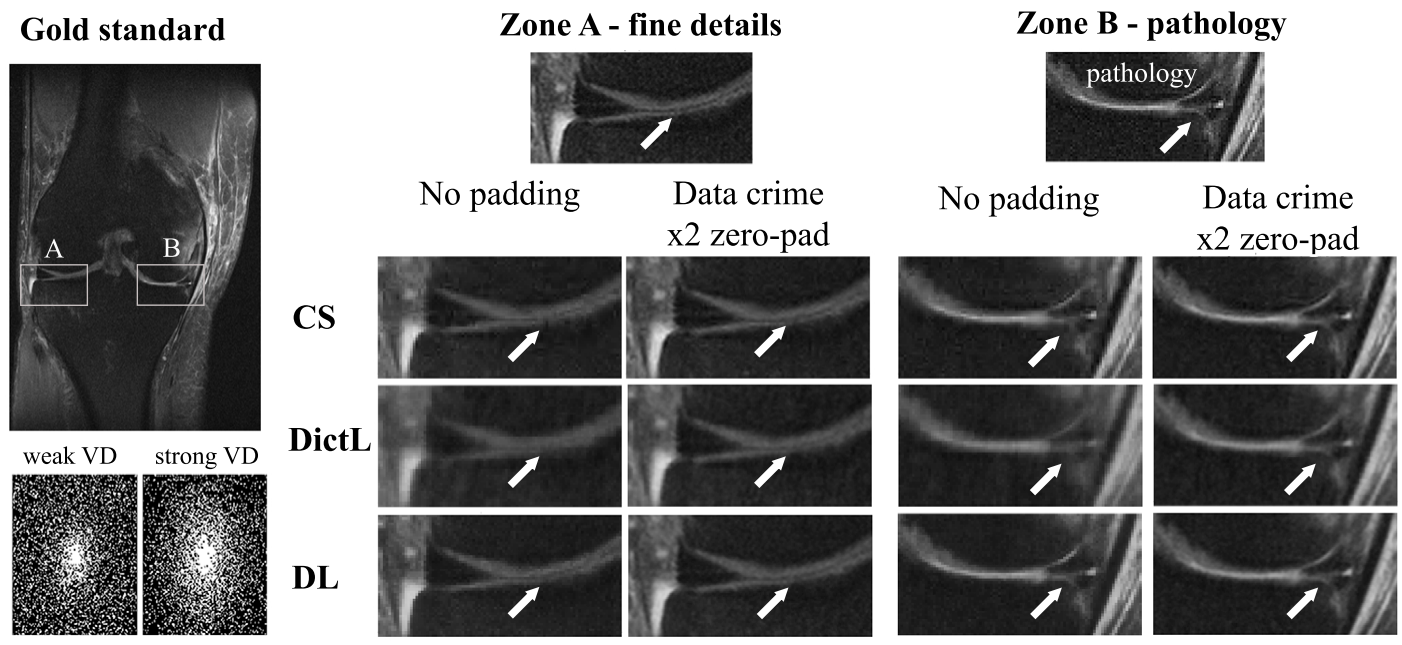
Data crime I. The CS, DictL, and DL algorithms were trained and tested using two versions of the same knee MRI dataset, one processed without zero padding and one with 2× zero padding. In the latter case, which represents the scenario of data crime I, the reconstructions exhibit sharper images with improved visibility of small, clinically relevant details. This illustrates that training inverse problem solvers using processed data may lead to overly optimistic results.

In the second experiment, we implemented the three algorithms and applied them to two versions of the same knee MRI dataset: one prepared without zero padding and the other prepared with 2× zero padding. The algorithms were trained on each dataset separately and then tested with the corresponding version of a test image that included fine details and a knee pathology (Fig. 4). As can be seen, all the algorithms produced sharper images in the data crime II scenario, where the data were zero padded: The fine details and the pathology became more visible than in the nonpadded case.

**Fig. 5.**
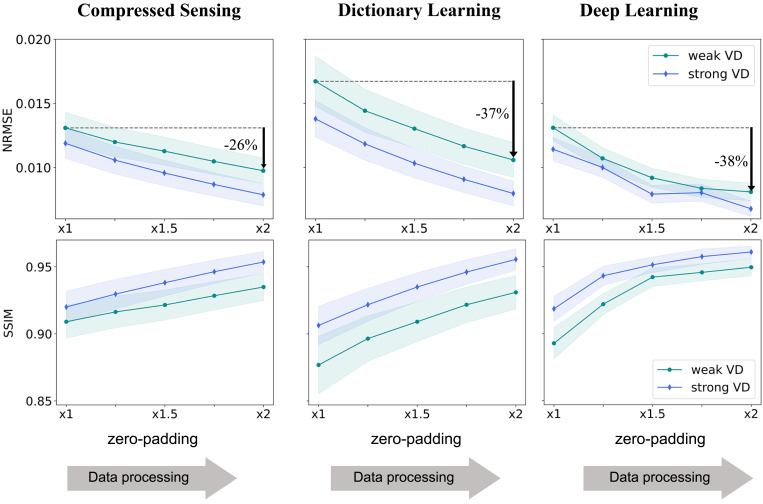
Data crime I statistics. The CS, DictL, and DL algorithms were trained and evaluated using data with various processing extents. The data-processing pipeline typically implemented inside commercial scanners includes k-space zero padding (Fig. 1*A*). Retrospective subsampling experiments were performed with VD subsampling with R=4. The curves display the mean and SD of the NRMSE and SSIM error metrics for the test set. Notice that both metrics show an artificial improvement that is correlated with the data-processing extent. This demonstrates that algorithms evaluated on retrospectively subsampled processed data tend to yield overly optimistic evaluation.

These results were further confirmed in a large set of experiments in which the algorithms were trained and tested on five versions of the underlying knee dataset representing five processing scenarios; each dataset contained 2,971 images. The hyperparameter calibration, training, and testing were performed for each dataset separately to optimize the algorithmic results for each processing scenario. We then computed the statistics of two image quality metrics, the NRMSE and structural similarity index (SSIM) (42), and plotted them against the zero-padding rate. Markedly, the results of the three algorithms exhibit the same behavior: Their NRMSE and SSIM values improve consistently with the zero-padding extent (Fig. 5). This improvement is completely artificial and stems only from data processing. Strikingly, for the 2× zero-padding case, which is often the default in commercial scanners, the NRMSE exhibits a large improvement of 26 to 42% (Table 1).

**Table 1.** Data crime I statistical results: The mean NRMSE and SSIM values measured for the test set

	NRMSE			SSIM		
	No padding	2× padding data crime	Artificial change	No padding	2× padding data crime	Artificial change
CS: weak VD	0.0131	0.0098	–26%	0.91	0.93	3%
CS: strong VD	0.0119	0.0079	–34%	0.92	0.95	4%
DictL: weak VD	0.0167	0.0106	–37%	0.88	0.93	6%
DictL: strong VD	0.0138	0.008	–42%	0.91	0.96	5%
DL: weak VD	0.0131	0.0081	–38%	0.89	0.95	6%
DL: strong VD	0.0114	0.0068	–41%	0.92	0.96	5%

All three algorithms yield overly optimistic results when trained and evaluated on zero-padded (processed) MRI data.

### Data Crime II.

To demonstrate the JPEG compression effect, we performed experiments in which the algorithms were trained and tested on different versions of the same underlying dataset. The JPEG compression level is determined by a quality factor (QF), where QF=75 is the default that yields lossy compression and values such as QF=50 and QF=20 yield increasing lossy compression (43). For reference, our experiments also include the case of image reconstruction from noncompressed data. In all cases, the hyperparameter calibration, algorithm training, and inference were made on the same type of data (i.e., with no compression or a specific QF).

**Fig. 6.**
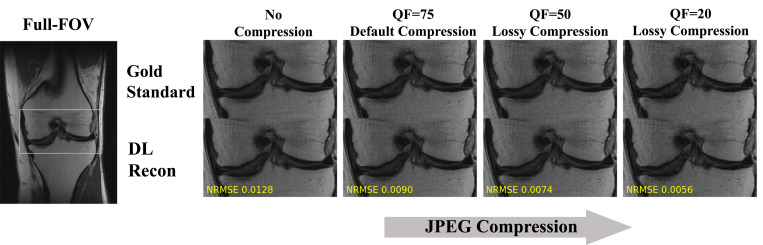
Example for data crime II. A DL algorithm was separately trained and tested on noncompressed data and different levels of JPEG-compressed data. Although the compression reduces the visual image quality, the NRMSE surprisingly reduces with increased compression, reflecting a seemingly better image quality. The reason is that in the retrospective experiments, both the gold standard and reconstructed images are based on processed data; hence, the error metric is blind to the processing and prone to bias. Strikingly, although the reconstructions from noncompressed and default-compressed data are visually similar, the NRMSE of the latter is lower by 30%. This demonstrates the implicit bias induced by training and evaluating algorithms on JPEG-compressed data.

In the first experiment, the DL algorithm was trained on the different datasets. Fig. 6 displays an example from the test set that shows the gold standard image and the DL reconstructions for data undersampled with *R* = 4. Generally, the visual quality of all the images (gold standard and reconstructed) reduces when the JPEG compression level increases (left to right in Fig. 6); this is expected from compressed data. However, the NRMSE metric shows an unexpected effect: It improves with the compression (i.e., the reconstruction error reduces, although the image’s visual quality degrades). The reason for this phenomenon is that in retrospective experiments, the reconstruction quality is measured with respect to a gold standard image that is based on the same underlying processed data. The error metrics, therefore, are blind to the data processing. Strikingly, the NRMSE could show a misleadingly large improvement even when the human eye cannot see any difference, as demonstrated in the left two columns of Fig. 6. Although the reconstructions from noncompressed and QF=75 data are visually similar, the NRMSE of the latter is 30% lower. This reflects the bias induced by data crime II.

**Fig. 7.**
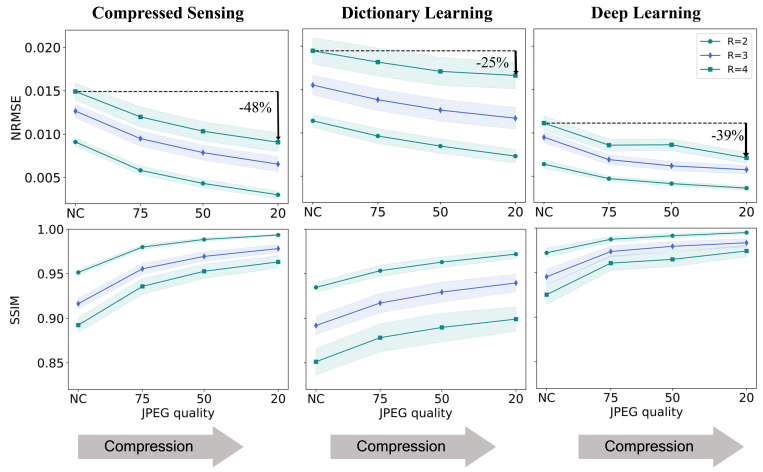
Statistical results demonstrating data crime II. The CS, DictL, and DL algorithms were applied to datasets with no compression and increasing JPEG compression levels. The graphs depict the mean and SD computed for the test set. Notice that all the curves show the same trend: The error metrics improve consistently with increased JPEG compression. This improvement is artificial and stems only from the data processing, which reduces the data entropy. The results, therefore, demonstrate the subtle bias caused by training inverse problem solvers on JPEG-compressed data.

The JPEG compression effect was further observed in a statistical analysis of an experiment in which the algorithms were trained and tested on the four types of data (noncompressed, QF = 75, QF = 50, and QF = 20) (Fig. 7). As illustrated, the error metrics exhibit a consistent improvement with the compression. Notably, this effect is systematically observed for all the studied algorithms and reduction factors (Table 2).

**Table 2.** Data crime II statistics: The mean NRMSE and SSIM values measured for the test set

	NRMSE			SSIM		
	No JPEG	JPEG QF	Artificial	No JPEG	JPEG QF	Artificial
	compression	20 data crimes	change	compression	20 data crimes	change
CS: *R* = 2	0.0091	0.0030	–67%	0.95	0.99	+4%
CS: *R* = 3	0.0127	0.0065	–48%	0.92	0.98	+7%
CS: *R* = 4	0.0149	0.0091	–39%	0.89	0.96	+8%
DictL: *R* = 2	0.0114	0.0073	–36%	0.93	0.97	+4%
DictL: *R* = 3	0.0155	0.0117	–25%	0.89	0.94	+5%
DictL: *R* = 4	0.0195	0.0166	–15%	0.85	0.90	+6%
DL: *R* = 2	0.0064	0.0036	–44%	0.97	0.99	+2%
DL: *R* = 3	0.0095	0.0058	–39%	0.95	0.98	+4%
DL: *R* = 4	0.0111	0.0071	–36%	0.93	0.97	+5%

All three algorithms yield overly optimistic results when trained and evaluated using JPEG-compressed data.

### Data Crimes Impact.

We now turn to experiments that aim to demonstrate a different phenomenon related to data crimes: poor generalization to unprocessed data when training on processed data.

**Fig. 8.**
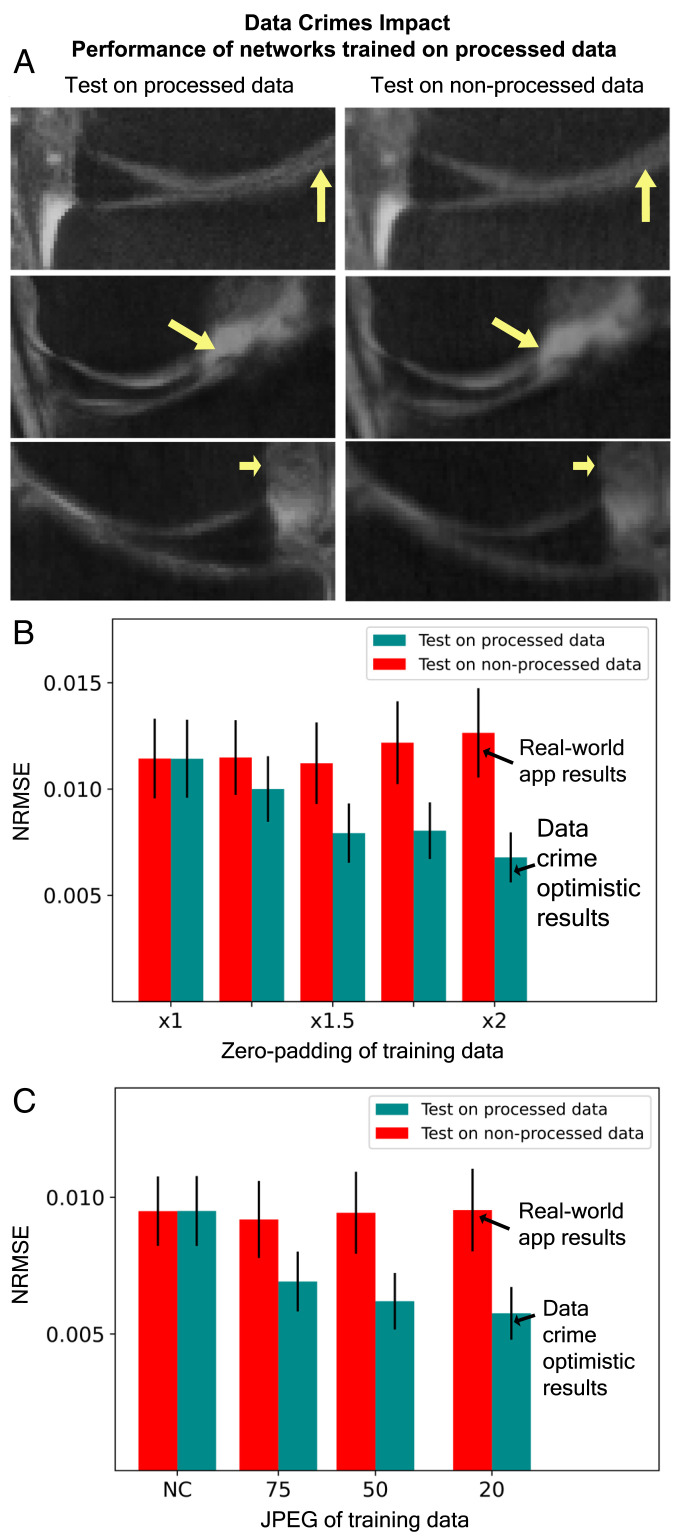
The potentially harmful impact of data crimes. In real-world applications, networks trained on processed data could be applied to unprocessed data. To show the negative effect, we took networks trained on processed data and tested them on both processed and unprocessed versions of the same test set. (*A*) Examples for knee MR images zoomed in on the clinically important meniscus area. Notice that in the real-world application (*Right*), the images are more blurred, and some details are missing (arrows). (*B*) Impact of data crime I. Notice that when networks trained on zero-padded data are applied to nonpadded data, their performance drops significantly, by up to 47%. (*C*) Impact of data crime II. Similarly, when networks trained using JPEG data are applied to non-JPEG data, their performance reduces.

In clinical translation research, algorithms trained using processed data eventually will be prospectively applied to unprocessed data. To demonstrate the negative consequence of this methodology, we conducted experiments in which networks trained on processed data were separately tested using processed and unprocessed versions of the underlying test set. We then compared their performance for those two cases. Fig. 8*A* shows three examples from knee MR images with zoom-in on the clinically important meniscus area. As can be seen, the images reconstructed from unprocessed data (Fig. 8 *A*, *Right*) are more blurred than are those reconstructed from the processed data (Fig. 8 *A*, *Left*), and some fine details are barely visible (arrows). This reduced performance is further illustrated in the statistical analysis shown in Fig. 8 *B* and *C*, where every pair of adjacent columns displays the performance of a single trained network. Clearly, when trained networks are applied to unprocessed data, their performance drops. This effect grows with the training data processing extent (left to right). Notice that in data crime I, where the network is trained on data processed with the default 2× zero padding but then applied to nonpadded data, its performance drops by 47% (Fig. 8*B*, rightmost pair of bars).

## Discussion

This study reveals that naive usage of open-access data in the development of MRI reconstruction algorithms could give rise to overly optimistic results. The underlying cause is that open-access data are commonly prepared with hidden processing pipelines that implicitly affect the data properties. Our study demonstrates that CS, DictL, and DL algorithms exhibit biased results for data prepared with common processing pipelines. Because this form of bias is largely unknown, it is frequently not addressed in the research literature. We coin the term data crimes to facilitate research in this field.

Our main observation is that bias stems from the unintentional coupling of hidden data-processing pipelines with later retrospective subsampling experiments. The data processing implicitly improves the inverse problem conditioning, and the retrospective subsampling enables the algorithms to benefit from that. This process may appear in different forms. In data crime I, the zero padding concentrates the true k-space data to the center. When VD sampling is later applied, those data are densely sampled. The increased amount of true data that become available to the algorithm makes the inverse problem easier to solve; hence, algorithms tend to exhibit misleadingly good results. In data crime II, the JPEG compression reduces the data entropy (i.e., it increases their sparsity and yields a more compact representation in a sparsifying transform domain). Modern reconstruction algorithms leverage sparsity priors or learn the compact representation from training data (6, 37, 44, 45); therefore, they benefit from the compression and yield biased results.

Another main insight from this study is that the error metrics might show a misleading evaluation in retrospective subsampling experiments. That occurs because they measure the difference between two images (the gold standard and reconstructed image) that are based on the same processed data. Ideally, the error metrics should measure the difference between the reconstructed image and the original unprocessed one. However, because the latter is unavailable (because it was not stored in the database), the metrics become blind to the data processing. As a result, they cannot reflect the true reconstruction quality and might produce misleading results.

Another phenomenon shown in our study is that networks trained on processed data do not generalize well to unprocessed data: They exhibit a significant performance drop (Fig. 8). Issues related to limited generalization of MRI reconstruction algorithms have been previously studied mainly in the context of distribution shifts (40, 46, 47). Here we show how limited generalization arises from unconscious off-label data usage. More importantly, in light of the large performance drop for real-world unprocessed data (Fig. 8), our experiments demonstrate the potentially harmful impact of data misuse in the development of clinically oriented algorithms.

In summary, this study reveals two types of algorithmic sensitivity related to misuse of publicly available processed data, in the context of MRI reconstruction: overly optimistic performance due to bias and limited generalization to unprocessed data. At present, there is growing interest in identifying sensitivities of such algorithms (5, 40, 48–51). However, recent studies focused mainly on investigating sensitivities with respect to adversarial attacks. Although these attacks are an important research tool, they are not observed in practice because MRI scanners are closed systems. Here, however, we focused on sensitivity related to a more common cause: off-label usage of public databases. While reviewing papers, we noticed that such usage is becoming increasingly more common due to the growing availability of public databases that offer various types of MRI data. Data crime I may be common because MR images found in public databases often are based on images produced by commercial scanners, in which the data-processing pipeline described in Fig. 1*A* is often applied by default. Additionally, data crime II may be common because JPEG images are highly prevalent: 73.3% of Internet websites contain JPEG-format data (52). These factors suggest that data crimes might be more common than intuitively expected.

While our research highlights two sources of bias related to data use, many other sources may exist and affect algorithmic results in published literature. As mentioned above, neglecting the complex-valued nature of MRI data and evaluating reconstruction algorithms on magnitude-only data improves the inverse problem conditioning and leads to overly optimistic results. Bias also may arise when some physical and physiological effects, such as contrast changes along echo trains, motion, or spatial phase variations, are not modeled in simulated data. Additionally, bias could arise from the choice of method used for computing ground truth images. For example, in parallel MRI, different methods for coil combination could lead to different results (53, 54). These issues are not limited to MRI. Bias also could arise from the usage of simulated or processed data in other applications that solve inverse problems, such as X-ray computed tomography (55), electrocardiography (56), bioluminescence tomography (57), microwave tomography (58), acoustic wave propagation inversion (59), and light curve inversion (60). These issues remain open for further research.

We suggest several guidelines that could help reduce data crimes. First, we strongly recommend that data curators provide a consolidated and comprehensive description of all data-processing steps. Second, for studies in which raw MRI data are not available and usage of processed data is necessary, we suggest the following: 1) Examine k-space and search for evidence of zero padding (see, for example, Fig. 2*C*). If such evidence is found, k-space can be cropped to remove these areas. 2) Report the data-processing steps in detail. By reporting them, studies based on processed data could be evaluated in the appropriate context. 3) Try to identify and estimate any bias that could stem from data processing by, for example, simulations (as in Figs. 5 and 7). Moreover, it is important to try to estimate any performance gap that could arise between implementation to processed and unprocessed data (see, for example, Fig. 8). Such estimations would further provide a more appropriate context for evaluating published results.

One argument often raised is that it is valid to compare several algorithms using processed data because we often are interested only in the relative performance of different techniques rather than an absolute measure. This is sometimes useful. However, if the limitations of the study are not reported correctly, then the results could negatively affect progress in the field because certain measures, such as the scan acceleration factor, are absolute and are compared across papers. For example, by using data subject to data crimes, one could claim good reconstruction quality from 10× acceleration, even if the effective acceleration without the crime was (for example) only 2×. The harm is that careful researchers may not be able to replicate such high accelerations on real data and, therefore, would find it more difficult to publish their work because it may not look competitive enough. Therefore, we suggest that any study comparing algorithms using synthetic or processed data include full disclosure of the data preparation pipelines.

It is worth mentioning that this work did not aim to benchmark the studied algorithms; instead, it aimed to show they all are affected similarly by the data crimes. However, as a side benefit, we did obtain benchmark comparisons. To ensure a fair comparison, we dedicated significant efforts to calibrating the hyperparameters of each algorithm for each processed version of the underlying dataset separately (*Materials and Methods*). Moreover, we ensured that the algorithms were calibrated, trained, and tested using identical datasets. We empirically observed that the studied algorithms perform overall at par, with an advantage of CS over DictL and a slight advantage of DL over both. However, due to the pipelines of the data crimes, all our computations were performed with single-coil magnitude, nonnegative images. The benchmarking of the algorithms for multicoil, complex-valued MRI data is beyond the scope of this work and remains for future research.

In summary, this research aims to raise a red flag regarding naive off-label usage of open-access data in the development of machine-learning algorithms. We showed that such usage may lead to biased results of inverse problem solvers. Furthermore, we demonstrated that training MRI reconstruction algorithms using such data could yield an overly optimistic evaluation of their ability to reconstruct small, clinically relevant details and pathology. This increases the risk of translation of biased algorithms into clinical practice. Therefore, we call for attention of researchers and reviewers: Data usage and pipeline adequacy should be considered carefully, reproducible research should be encouraged, and research transparency should be required. Through this work, we hope to raise community awareness, stimulate discussions, and set the ground for future studies of data usage.

## Materials and Methods

### Raw Data.

To demonstrate the effects of the hidden processing pipelines, we took raw MRI data and spoiled them with carefully controlled processing steps. The raw data were obtained from the training and validation sets of the FastMRI database (18), in which fully sampled ground truth data are available (the FastMRI test set was not used because it contains only undersampled data). This section describes the raw datasets; the processing steps were described in the main part of the paper for each data crime separately.

1. We first used brain data. In the experiment presented in Fig. 3, we used a single 320 × 320 brain image.
2. Next, we used knee fat-saturated proton density (FSPD) data. In the knee pathology experiments (Figs. 4 and 8*A*), we used data from multicoil FSPD scans because knee pathology is usually observed in this type of MRI scan. The training set consisted of 2,849 randomly chosen slices obtained from 300 subjects, and the test cases were taken from two images that contain pathological details (found in FastMRI files named file1000425.h5 and file1002455.h5).
3. Finally, we used knee proton density (PD) data. In the experiments that were used for demonstration and statistical analysis of the two data crimes (Figs. 5–8), we used 640 × 372 slices obtained from multicoil PD scans. Specifically, we used 1,427 slices obtained from 80 subjects for training and 122 slices obtained from seven subjects as the test set. All the slices were chosen randomly.

When constructing the knee PD and FSPD datasets, we used only slices from central anatomical regions (i.e., edge slices that contain mostly noise were removed). Additionally, for each dataset, we chose 10 random slices obtained from 10 different subjects and reserved them for tuning the hyperparameters of the studied algorithms; these slices were not included in the training or test sets. It is worth mentioning that the limited number of slices used for hyperparameter calibration was dictated by the need to perform vast computations over a huge search space, especially for the DictL algorithm, as described in *DictL Algorithm*.

### Experimental Overview.

We designed our research framework to enable isolating the bias related to the data crimes in a controlled setup. Additionally, because a side result of this study was the benchmarking of the studied algorithms, we also dedicated significant efforts to ensuring their fair comparison. Here we detail the steps that were taken for these two aims.

First, to mimic a scenario in which users download a dataset from an online resource and then optimize the parameters of their algorithm for that specific dataset, we prepared separate processed datasets for each instance of the data-processing parameters (i.e., for each zero-padding factor or JPEG QF) and ensured there was no mixture between the datasets. We then calibrated, trained, and tested the algorithms on each processed dataset separately. This ensured that each algorithm was evaluated using instance-optimal parameters; therefore, it mitigated bias related to hyperparameter tuning. Second, we applied the three algorithms to identical datasets; their results, therefore, are comparable. Finally, we generated sampling masks on the fly (i.e., a different random mask was generated for each k-space example during the training and test sessions). This technique enables generating a large number of sampling masks while maintaining their statistics; hence, it prevents overfitting to any particular sampling mask.

### Intensity Scaling.

The intensity of the processed images was normalized by dividing the values of each image by their 98th percentile. This is a practical data normalization technique often used in DL studies because neural networks are highly suited for input values in the range of [0, 1].

### Sampling.

In the retrospective subsampling experiments, we generated random two-dimensional (2D) subsampling masks from predefined probability density functions (PDFs) using Monte Carlo experiments. We implemented three subsampling schemes: 1) random uniform, in which the PDF was constant and equal to 1/R (*R* is the acceleration factor); 2) weak VD, in which the PDF was constructed by the function $f(r)={\left(1-r\right)}^{p}$, where *r* is the distance from the k-space center and *p* is the power (37), which was set to *p* = 7 in this case; and 3) strong VD, in which the PDF also was constructed by $f(r)={\left(1-r\right)}^{p}$ and the power was set to *p* = 1, *p* = 2, and *p* = 3 for reduction factors of *R* = 2, *R* = 3, and *R* = 4 correspondingly. All the sampling masks included a small, fully sampled area in the center of the k-space. In parallel imaging, this area is often known as the calibration region (16). In single-coil MRI experiments, this region ensures sampling of the low-frequency data and helps stabilize the computational results. The calibration region size was 12 × 7 pixels for the 640 × 372 knee images and 6 × 6 pixels for the 320 × 320 brain image. In the zero-padding experiments, where the image size varied, the calibration region size scaled with the image size.

### Algorithms.

The CS, DictL, and DL algorithms recover an MR image from subsampled k-space measurements by solving an inverse problem that has the following general form:

[1] $$\hat{x}=\arg\underset{x}{\min}\frac{1}{2}{\left\|Ex-y\right\|}_{2}^{2}+\lambda R(x),$$

where **x** is the image to be reconstructed, **y** are the k-space measurements, **E** is an encoding operator that describes the imaging system, R(x) is a regularization term, and λ is a trainable parameter that controls the tradeoff between the data consistency (DC) term (the first term in Eq. **1**) and the regularization term. In MRI, the encoding operator **E** is typically described as E=UF, where **F** is the Fourier transform and **U** is an operator that describes the k-space subsampling. The studied algorithms differ in their regularization terms and optimization techniques, as described next.

#### CS algorithm.

This algorithm formulates Eq. **1** as a convex optimization problem with an $l_{1}$ prior that promotes the sparsity of **x** in a sparsifying transform domain (37). A common choice for the prior is an $l_{1}$-wavelet one; the optimization problem is then

[2] $$\hat{x}=\arg\underset{x}{\min}\frac{1}{2}{\left\|Ex-y\right\|}_{2}^{2}+\lambda ||\Psi x|{|}_{1},$$

where Ψ is the wavelet transform. Eq. **2** can be solved using different optimization techniques; here it was solved using the fast iterative shrinkage-thresholding algorithm (61). Our implementation was based on the SigPy python toolbox (62).

The CS algorithm has one tunable parameter, λ. We calibrated it through a grid search, in which the grid included values in λ∈[1e−9,1e−1]. We ran the grid search over 10 images from a subset of the data that were reserved for hyperparameter tuning. We then computed the mean NRMSE over those 10 images, and the value of λ that corresponded to the lowest mean NRMSE was chosen. Because in the experiments of data crime I the image size varied with the zero padding, we repeated this procedure for each image size separately. However, we empirically observed that the same λ value was chosen for all image sizes. The chosen values were λ=0.005 for the brain data (Fig. 3) and λ=0.001 for the knee data (Figs. 4–8).

#### DictL algorithm.

The DictL algorithm reconstructs the image **x** by jointly learning an image domain patch dictionary **D** and a sparse code **A**. The dictionary, which is used for reconstruction of patches, is a sparsifying transform that is learned directly from the subsampled k-space data. In this method, the image is reconstructed by representing it as a sparse linear combination of dictionary atoms, x=DA. The DictL algorithm jointly solves for the image **x**, the dictionary **D**, and the sparse code **A** (38). The algorithm adaptively learns the dictionary from the subsampled k-space while reconstructing the image; that is, it is trained without any other examples or access to the fully sampled k-space data. The learning is done over image patches.

The optimization problem is formulated as follows:

[3] $$\begin{array}{ll} \underset{x,A,D}{\min}\frac{1}{2}||Ex-y|{|}_{2}^{2}+\frac{\lambda_{D}}{2}||x-R(DA)|{|}_{2}^{2}\; & \\ \;\;\;\;\;\;\;\;\;\;\;\;\;\;\;\;\;\;\;\;\;\;\;\;\;\;\;\;\;\;\;\;\;\;\;\;\;\;\;\;\;\;\;\;\;\;\;\;\;\;\;\;\text{subject}\;\text{to}\;||a_{l}|| & \leq K,\;l=1,\ldots ,L\\ \;\;\;\;\;\;\;\;\;\;\;\;\;\;\;\;\;\;\;\;\;\;\;\;\;\;\;\;\;\;\;\;\;\;\;\;\;\;\;\;\;\;\;\;\;\;\;\;\;\;\;\;\;\;\;\;\;\;\;\;\;\;\;\;\;\;\;\;\;\;\;\;\;\;||d_{p}|{|}_{2} & \leq 1,\;p=1,\ldots ,P, \end{array}$$

where **R** is a reshaping operator that reshapes patches into an image; $a_{l}$ are the columns of **A**, where each column is a vectorized patch from the image; *K* is the sparsity level; *L* is the number of patches that are used as training examples during one iteration of the algorithm; $d_{p}$ are the columns of the dictionary **D**, where each column is a vectorized atom; and *P* is the number of dictionary atoms.

We implemented the DictL algorithm in Python using our open-source code (63). The algorithm solves Eq. **3** through alternating minimization, with K-SVD (64) for the dictionary update and orthogonal matching pursuit (65) for the sparse code update.

We dedicated vast computations for calibrating the five tunable hyperparameters of the DictL algorithm, which are *P*, *K*, *λ_D_*, block size *b* (the blocks are symmetric, i.e., if *b* = 8, then the block size is 8 × 8), and the number of outer iterations of the alternating minimization algorithm, *N_iter_*. Due to the varying image size in the zero-padding experiments, we repeated the calibration process for each image size separately; this added another dimension. Furthermore, we repeated the search for a set of five images that were reserved for hyperparameter tuning (the number of images was dictated by the need for vast computations). Altogether, we trained the DictL algorithm five times for each combination of the six parameters; this resulted in the evaluation of 77,686 DictL instances. For each combination, we computed the mean NRMSE over the five images and chose the hyperparameter values that produced the lowest NRMSE. This set of computations was highly time consuming; it was conducted on 200 central processing units (CPUs) in parallel for over 4 wk. The tested grid search values were P∈[100,200,300], K∈[5,13] where the step size was 2, $\lambda_{D}\in [1e-5,1e-4,1e-3,1e-2,1e-2],\;b\in [4,32]$ where the step size was 4, and $N_{\mathrm{iter}}\in [5,13]$ where the step size was 2. The chosen hyperparameter values are detailed in *SI Appendix*.

#### DL algorithm.

We studied the model-based reconstruction using deep learned priors (MoDL) algorithm, which gives state-of-the-art performance in MRI reconstruction (39). The MoDL algorithm solves the following optimization problem:

[4] $$\hat{x}=\arg{\underset{x}{\min}}_{}\frac{1}{2}{\left\|Ex-y\right\|}_{2}^{2}+\lambda {\left\|x-D_{w}(x)\right\|}^{2},$$

where $D_{w}(x)$ is the output of a convolutional neural network (CNN). This optimization problem is solved using an unrolled deep neural network that includes interleaved CNNs and DC blocks. The DC blocks ensure consistency of the solution with the k-space measurements; the back-propagation through them is implemented using the conjugate gradient (CG) algorithm (66). The MoDL unrolled network is trained in an end-to-end supervised manner where the input is an aliased image obtained from the zero-filled subsampled k-space data, and the target is a “gold standard” image obtained from the fully sampled k-space.

In our implementation, the network architecture included six unrolls, CNNs with a U-net structure (67), weight sharing, and eight CG steps in the DC blocks. The training was performed using an *l*1-loss and the Adam optimizer (68) with gradient accumulation such that the effective batch size was 20. The number of epochs was 70. We implemented MoDL using PyTorch (69).

### Data Crime I Experiments.

In this section, we provide implementation details regarding the experiments performed to demonstrate the effects of data crime I. In the first experiment, which demonstrates the difference between global and effective sampling (Fig. 2), a set of 15 random masks was generated for each combination of a subsampling scheme and zero-padding factor. The curves in Fig. 2 depict the mean effective rates measured over those sets.

In the next experiments, which demonstrate the zero-padding effect (Figs. 3–5), we implemented VD sampling with an acceleration factor of *R* = 4. In these experiments, the MoDL network could not be trained on full-size images because the zero padding enlarges the image size to an extent that poses a computational challenge even with modern graphics processing units (GPUs). However, a major advantage of MoDL is that it is convolutional and at inference can be implemented to any image size (39). Therefore, we trained MoDL on patches extracted from training images. The patch size was 0.25 of the image size in each dimension, and a single patch was extracted randomly from each image. We also computed the k-space of the patch and used it in the MoDL DC blocks. In contrast, the network was applied to the full-size test images during inference; therefore, the results shown in Fig. 5 represent the reconstruction error for full images.

### Data Crime II Experiments.

In the second set of experiments, we studied how JPEG compression of the underlying data influences the performance of reconstruction algorithms. We prepared the processed datasets using the standard JPEG implementation found in the Pillow library (70). In the JPEG experiments, the reduction factor ranged from *R* = 2 to *R* = 4 (Table 2).

### Data Crimes Impact Experiments.

To show the negative impact of applying networks trained on processed data to unprocessed data, we used networks that were trained in the experiments described in *Results*. Then we performed inference using the unprocessed versions of each dataset, which also were described in *Raw Data*. Specifically, for Fig. 8*A*, we used networks trained on FSPD data as described for Fig. 4. Additionally, for Fig. 8 *B* and *C*, we used networks trained for the statistical experiments shown in Figs. 5 and 7, respectively.

### Image Quality Metrics.

We quantified the data crimes’ effects by studying how the data-processing pipelines influence two highly common image quality metrics: the NRMSE and the SSIM (42); the latter was implemented using the SSIM-python imaging library (PIL) library (71).

### Computational Overview.

This research involved extensive computations due to the need to optimize, train, and test each algorithm on each processed version of the underlying dataset. The hyperparameter tuning required about 1 mo of computations, and the experiments required another month. Altogether, the compute time was about 2 mo using 200 CPUs and 12 GPUs. All our experiments were performed on 12GB Nvidia Titan Xp GPUs and Interl(R) Xeon(R) Silver 4116 CPUs.

## Supplementary Material

Supplementary File

## Data Availability

All the original code for this study and the weights of the pretrained networks have been deposited in Zenodo, https://zenodo.org/record/6015698#.YiJDNBPMJqs (72). The raw magnetic resonance imaging data used for this study is available in the fastMRI database, https://fastmri.org/ (18).
