# Supplementary material for: Implicit data crimes: Machine learning bias arising from misuse of public data
Source: Proc Natl Acad Sci U S A. 2022 Mar 21;119(13):e2117203119. doi: 10.1073/pnas.2117203119 (PMC9060447; doi:10.1073/pnas.2117203119)
Supplement: Supplementary File [file pnas.2117203119.sapp.pdf]

PNAS
www.pnas.org

Supplementary Information for

Implicit Data Crimes: Machine Learning Bias Arising from Misuse of Public Data

Efrat Shimron, Jonathan I Tamir, Ke Wang and Michael Lustig

Corresponding author: Efrat Shimron.
E-mail: efrat.s@berkeley.edu

This PDF file includes:

Supplementary text

Supporting Information Text

Materials and Methods

In this section we provide details regarding the hyperparamter tuning of DictL algorithm and the chosen optimal paramters.

The DictL algorithm has five tunable paramters: the number of dictionary atoms P, sparsity level K, block size b, regularization parameter λ_D, and the number of outer iterations of the alternating minimization algorithm, denoted here by N_{iter}. In addition, the image size varied in the zero padding experiments, hence the grid search was repeated for each image size. The basic image size was 640×372, and the grid search was repeated for padding ratios of 1 (i.e. no padding), 1.25, 1.5, 1.75 and 2. We also repeated the grid search for weak VD and strong VD sampling schemes. The chosen values were as follows:

- No padding, weak VD: $\lambda_D = 1e-5$, P=300, K=11, N_{iter}=13.
- 1.25x padding, weak VD: $\lambda_D = 1e-3$, P=200, K=11, N_{iter}=13.
- 1.5x padding, weak VD: $\lambda_D = 1e-2$, P=300, K=9, N_{iter}=11.
- 1.75x padding, weak VD: $\lambda_D = 1e-3$, P=300, K=11, N_{iter}=13.
- 2x padding, weak VD: $\lambda_D = 1e-3$, P=200, K=11, N_{iter}=13.
- No padding, strong VD: $\lambda_D = 1e-5$, P=300, K=11, N_{iter}=11.
- 1.25x padding, strong VD: $\lambda_D = 1e-4$, P=200, K=11, N_{iter}=13.
- 1.5x padding, 1.75x padding and 2x padding, strong VD: $\lambda_D = 1e-3$, P=200, K=11, N_{iter}=13.

We empirically observed that the same block size, $b = 16$, was chosen for all cases. Additionally, the number of training patches used in each outer iteration of the algorithm was fixed and set to $L = 500$.
